# The standing fixed flexion view detects narrowing of the joint space better than the standing extended view in patients with moderate osteoarthritis of the knee

**DOI:** 10.3109/17453674.2010.483989

**Published:** 2010-05-21

**Authors:** Tuukka Niinimäki, Risto Ojala, Jaakko Niinimäki, Juhana Leppilahti

**Affiliations:** ^1^Departments of Orthopaedic and Trauma Surgery; ^2^Diagnostic Radiology, Oulu University Hospital, OuluFinland

## Abstract

**Background and purpose:**

It is unclear whether osteoarthritis (OA) of the knee is seen better in standing flexion position radiographs than in the standing extended view. We assessed the value of standing flexion views.

**Patients and methods:**

We retrospectively evaluated 1,090 radiographs of 545 consecutive knees with non-traumatic knee pain, comparing standing fixed flexion view (FFV) and standing extended view (SEV). OA was classified according to the Kellgren-Lawrence (KL) radiographic grading scale and joint space widths were measured.

**Results:**

Medial joint space width was lower on average in the FFV, with the greatest difference in KL II knees. Medial full-thickness loss of cartilage was also seen more often in the FFVs of knees with moderate OA (KL II–III) than in the SEVs (6% vs. 19%).

**Interpretation:**

Using FFV, there is no need to measure the exact knee flexion angle to use fluoroscopy. In earlier studies, the FFV has been found to be reproducible and easy to use in clinical practice. We recommend using flexion views when deciding the appropriate type of intervention in patients with OA. Full-thickness loss of cartilage in particular is better seen in the flexion view, which may be helpful if planning unicompartmental knee arthroplasty.

## Introduction

The standing extended view (SEV) is the common radiographic examination for evaluation of knee OA. However, it has been reported that narrowing of the joint space is better visualized in fixed flexion views (FFVs), which have also been found to be reproducible and easy to use in clinical practice (Piperno et al. 1998, Vignon et al. 2003, Duddy et al. 2004). However, one report has claimed that narrowing of the joint space width in flexed knee radiographs is not always caused by OA (Deep et al. 2003) and there is still disparity regarding the optimal knee flexion angle (Bhatnagar et al. 2006).

We assessed the value of the FFV in clinical practice and tried to find out whether there are groups of patients for whom the flexion view would be beneficial.

## Patients and methods

We retrospectively evaluated radiographs of both knees in 590 consecutive patients (264 men and 326 women, 2,360 radiographs), obtained between Oct 2006 and Sep 2007. Exclusion criteria were an earlier knee arthroplasty or intra-articular fracture, missing or poor visualization of the calibration disc, poor quality of the radiograph, or lack of either the SEV or the FFV radiograph. The main causes for exclusion were absence of the calibration disc, which was not in routine use at the time of this study, and earlier knee replacement. Eventually, 545 knees (301 patients) and 1,090 radiographs were included. Most patients were middle-aged or elderly and all suffered from OA. Their mean age was 60 (18–92, SD 14) years.

Prior to the physical examination, both SEV and FFV had been taken bilaterally with a digital radiography system in every case, and an additional standing extended lateral view had been taken of the symptomatic knee. The SEV had been taken with the patient standing upright in front of the film cassette, facing the X-ray beam with the backs of the thighs touching the cassette. The beam was aimed horizontally at the joint line. The FFV had been taken with the feet in 10° external rotation, and toes touching against the vertical table. Additionally, the subject was asked to bend the knees until the anterior surface of knees and thighs leaned against the table. The X-ray beam was angled without fluoroscopy to 10° pointing caudal. For further measurements, a metal calibration disc 30 mm in diameter was attached to the medial side of the knee at the level of the epicondylar axis (Figure).

**Figure:**
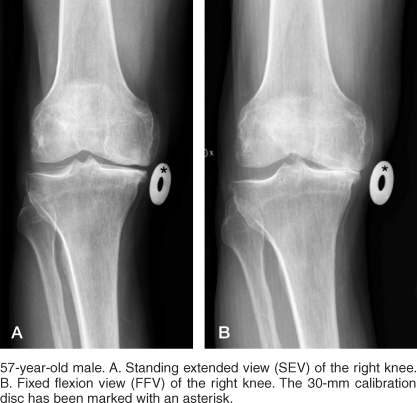
57-year-old male. A. Standing extended view (SEV) of the right knee. B. Fixed flexion view (FFV) of the right knee. The 30-mm calibration disc has been marked with an asterisk.

The digital radiographs were calibrated and read separately. Medial and lateral joint space widths (MJSW and LJSW) were measured and OA was classified blindly according to the clinical data (Kellgren-Lawrence (KL) classification) (Table 1).

**Table 1.** The Kellgren-Lawrence classification (Kellgren and Lawrence 1957)

Grading	Description
KL I	Doubtful narrowing of joint space and possibly osteophytic lipping
KL II	Definite osteophytes and possible narrowing of joint space
KL III	Moderate multiple osteophytes, definite narrowing of joint space and some sclerosis, and possible deformity of bone ends
KL IV	Large osteophytes, marked narrowing of joint space, sever sclerosis, and definite deformity of bone ends

### Statistics

Analysis of variance (ANOVA) was used to compare more than 3 groups, or the Kruskal-Wallis test in the case of non-normally distributed data. Comparisons between 2 groups were performed using Student’s t-test or the Mann-Whitney U test, the latter for data that were not normally distributed. SPSS software version 16.0 was used.

## Results

On average, joint space widths were lower in the FFV than in the SEV, the differences being higher on the medial side (Table 2).

**Table 2.** Differences in joint space width between the standing extended view (SEV) and fixed flexion view (FFV)

	Medial (SEV–FFV)	Lateral (SEV–FFV)	p-value
Men	0.8 (1.0)	0.2 (1.0)	< 0.001
Women	0.7 (0.9)	0.2 (0.9)	< 0.001
Total	0.7 (1.0)	0.2 (1.0)	< 0.001

Average values (SD) in mm.

In the medial side, joint space widths were lower in the FFV in all degrees of arthritis (Table 3), but in the lateral side there was no difference in joint space width in the knees with mild arthritis (Table 4).

**Table 3.** Kellgren-Lawrence classification of arthritis, medial joint space width (MJSW) on the standing extended view (SEV), and the difference in MJSW between the fixed flexion view (FFV) and the SEV

Classification (SEV)	No. of knees (%)	MJSW on SEV	MJSW difference ^**a**^
No arthritis	72 (13)	5.4 (0.9)	0.6 (0.7)
KL I	225 (41)	4.9 (1.1)	0.6 (0.8)
KL II	136 (25)	4.3 (1.4)	1.0 (1.1)
KL III	79 (15)	3.2 (2.0)	0.9 (1.1)
KL IV	33 (6)	2.5 (2.1)	0.7 (0.9)
Total	545 (100)	4.4 (1.6)	0.7 (1.0)

Average values (SD) in mm.

^**a**^ Difference in MJSW between SEV and FFV views.

**Table 4.** Kellgren-Lawrence classification of arthritis, lateral joint space width (LJSW) on the standing extended view (SEV), and the difference in LJSW between the fixed flexion view (FFV) and the SEV

Classification (SEV)	No. of knees (%)	LJSW on SEV	LJSW difference ^**a**^
No arthritis	72 (13)	5.9 (1.2)	–0.1 (0.8)
KL I	225 (41)	5.6 (1.1)	0.0 (1.0)
KL II	136 (25)	5.7 (1.7)	0.2 (1.1)
KL III	79 (15)	6.1 (2.1)	0.4 (1.1)
KL IV	33 (6)	5.4 (3.0)	0.7 (1.2)
Total	545 (100)	5.7 (1.6)	0.2 (1.0)

Average values (SD) in mm.

^**a**^ Difference in LJSW between SEV and FFV views.

In the FFV, medial full-thickness loss of cartilage surface (MJSW < 1 mm) was seen in 40 (19%) of the 215 knees with moderate OA (KL II–III). In the SEV, the corresponding number of knees was 12 (5.6%). Corresponding figures for the lateral side were 12 (5.6%) and 3 (1.4%), respectively.

The most common change in KL classification was seen in those assigned to KL II on the basis of the SEV findings. According to the FFV, 14% of these knees were classified as KL III (Table 5).

**Table 5.** Kellgren-Lawrence (KL) classification according to the SEV view and the FFV view

SEV, KL-classification	FFV view, KL-classification				
	NA ^**a**^	1	2	3	4
NA**^a^** (%)	68 (94)	4 (6)	0	0	0
1	2 (1)	206 (92)	17 (8)	0	0
2	0	1 (1)	115 (85)	20 (15)	0
3	0	0	0	76 (96)	3 (4)
4	0	0	0	0	33

^**a**^ NA = no radiographic arthritis.

## Discussion

On average, OA was classified as being more severe on the basis of the FFV. Variability in MJSW was also seen in the case of knees with no radiographic OA, where the MJSW was 0.6 mm lower on average in the FFV than in SEV. In this respect, our results agree with those of an earlier study and point to natural variability in MJSW possibly caused (at least in part) by variations in the thickness of the articular cartilage between different parts of the knee and sliding of the knee joint during flexion (Deep et al. 2003, Patel et al. 2004).

We could not repeat these results on the lateral side, however. In non-arthritic knees, the LJSW was actually 0.1 mm higher in the FFV than in SEV while there was no difference between the two radiographs (LJSW = 0.0 mm (SD 1.0)) in the cases with mild arthritis (KL I). The explanation for this different result on the lateral side may lie in a difference in shape between the medial and lateral tibial plateau.

The strength of this study lies in its large number of consecutive patients. Radiographic measurement of joint space width was also designed to be as accurate as possible. The absence of any calibration disc may have caused measurement errors in the earlier studies (Mazzuca et al. 2004).

Since the indication for major surgery for OA is pain with radiographic findings of severe OA, patients with KL II or III arthritis benefit from having FFV taken. The degree of OA can change from moderate to severe between the SEV and the FFV. In particular, medial full-thickness loss of cartilage, which may be an indication for unicompartmental knee arthroplasty (Goodfellow et al. 2006, Pandit et al. 2006), was seen more often in the FFV. These findings are helpful in choosing the best treatment option for each patient, especially in cases with a painful knee but only mild or moderate radiographic OA according to the SEV.

In earlier studies the FFV has been found to be reproducible and easy to use in clinical practice, and when using FFV there is no need for measurement of the precise knee flexion angle or for the use of fluoroscopy.

In conclusion, we recommend routine use of the FFV if planning surgery for patients with OA in order to obtain a better picture of the thickness of the cartilage and the severity of arthritis in the knee. This is especially beneficial for patients for whom it is difficult to choose whether the best surgical treatment option is osteotomy, unicompartmental arthroplasty, or total knee arthroplasty. In the evaluation of medial joint space narrowing in young patients and non-arthritic knees, one should be aware of the natural variability in MJSW, especially if there are no other signs of arthritis (osteophytes or subchondral sclerosis).
